# The Role of Insulin-Like Growth Factor System in Soft Tissue Sarcomas: From Physiopathology to Targeted Therapeutic Approaches

**DOI:** 10.1080/13577149878028

**Published:** 1998-06

**Authors:** Walter Zumkeller

**Affiliations:** 1 Department of Hematology/Oncology Children's University Hospital Heidelberg Germany; 2 German Cancer Research Centre Division of Cytogenetics Im Neuenheimer Feld 280 Heidelberg 69120 Germany

## Abstract

*Purpose/Results.* Although surgical, chemo- and radiotherapeutic treatment regimens in patients with soft tissue sarcomas have constantly been refined over the past two decades, the survival rate for these patients is rather low.

*Discussion.* There is a great need to investigate the mechanisms for oncogenesis and to identify the factors involved in malignant transformation in sarcomas. Among these factors, IGFs are thought to play a pivotal role as progression factors in various types of sarcomas. The dysregulation of the IGF-II synthesis, e.g. by loss of imprinting which occurs in most types of sarcomas, is a permissive effect through the suppression of cell death. In addition, cells that overexpress the type *I IGF receptors* are more susceptible to transformation by oncogenes. As *TP53* suppresses the activity of IGF-II P3 and P4, as well as the type I IGF receptor promoter, mutations of *TP53* in sarcomas may alternatively lead to the activation of these factors. Finally, the phenomenon of non-islet cell tumour hypoglycaemia that occurs in patients with sarcomas, and which is related to the secretion of IGF-II prohormones, is discussed. Future therapeutic strategies may be based upon the application of antibodies or antisense oligonucleotides directed against the type I IGF receptors, with the common goal of inducing apoptosis in sarcoma cells. Ultimately, these and other therapeutic approaches may lead to a better outcome in patients suffering from sarcoma.


*Sarcoma* (1998) **2**, 69–76

REVIEW

### The role of insulin-like growth factor system in soft tissue sarcomas: from physiopathology to targeted therapeutic approaches

WALTER ZUMKELLER

*Department of Hematology/Oncology, Children's University Hospital, Heidelberg, Germany*

**Abstract**

*Purpose/Results.* Although surgical, chemo- and radiotherapeutic treatment regimens in patients with soft tissue sarcomas have constantly been refined over the past two decades, the survival rate for these patients is rather low.

*Discussion.* There is a great need to investigate the mechanisms for oncogenesis and to identify the factors involved in malignant transformation in sarcomas. Among these factors, IGFs are thought to play a pivotal role as progression factors in various types of sarcomas. The dysregulation of the IGF-II synthesis, e.g. by loss of imprinting which occurs in most types of sarcomas, is a permissive effect through the suppression of cell death. In addition, cells that overexpress the type *I IGF receptors* are more susceptible to transformation by oncogenes. As *TP53* suppresses the activity of IGF-II P3 and P4, as well as the type I IGF receptor promoter, mutations of *TP53* in sarcomas may alternatively lead to the activation of these factors. Finally, the phenomenon of non-islet cell tumour hypoglycaemia that occurs in patients with sarcomas, and which is related to the secretion of IGF-II prohormones, is discussed. Future therapeutic strategies may be based upon the application of antibodies or antisense oligonucleotides directed against the type I IGF receptors, with the common goal of inducing apoptosis in sarcoma cells. Ultimately, these and other therapeutic approaches may lead to a better outcome in patients suffering from sarcoma.

**Key words:** *insulin-like growth factors, IGF-binding proteins, soft tissue sarcoma, Ewing's sarcoma, rhabdomyosarcoma, tumour hypoglycaemia.*

### Introduction

Steady progress has been made in the identification of genetic alterations and prognostic factors in sarcomas. However, survival rates of patients with sarcomas remain unsatisfactorily low in spite of aggressive treatment involving highly toxic multi-drug chemotherapeutic as well as radiotherapeutic regimens. However, considerable progress has been made in the past decade in identifying important factors involved in tumour growth. In addition, the factors involved in the induction and modulation of apoptosis, among them IGFs, are of particular interest. IGF-I and IGF-II are mitogenic polypeptides and are synthesized by most normal and malignant tissues, where they are involved in autocrine and/or paracrine types of action. The IGF system is further complicated by the presence of specific IGF-binding proteins (IGFBP-1 to −7) which bind IGFs with high affinity. These IGFBPs are thought to play an important role in modulating IGF responsiveness in normal as well as malignant cells. The biological effects of IGFs are effected through the insulin, type I IGF (IGF-I) and type II (IGF-II) receptors. The type I IGF receptor in particular is involved in growth, differentiation and inhibition of apoptosis depending on activation by IGFs.[1] Rescue from cell death by IGF-I is mediated through this receptor, and antibodies against this receptor can block the rescuing function of the growth factor.[2] Moreover, IGF-I had no effect in preventing etoposide-induced apoptosis in fibroblasts derived from mice embryos that have a targeted disruption of the *type I IGF receptor*.[3]

The autocrine growth hypothesis states that both normal and malignant cells can synthesize and secrete polypeptide growth factors that will bind to their own cell surface receptor and stimulate cell proliferation.[4] *IGFs* are abnormally expressed in some paediatric solid tumours, and certain tumours are responsive or dependent upon IGFs for proliferation. In addition, many tumour cell lines express IGFs as autocrine factors and IGFBPs, which, in

Correspondence to: W. Zumkeller, German Cancer Research Centre, Division of Cytogenetics, Im Neuenheimer Feld 280, 69120 Heidelberg, Germany. Tel: +49 6221 423271; Fax: +49 6221 423277; E-mail: w.zumkeller@dufz-heidelberg.de.

1357-714X/98/020069-08 ©1998 Carfax Publishing Ltd turn, regulate the bioavailability and bioactivity of IGFs.[5–7] The imprinted genes *IGF-II* and *H19* are expressed during embryonal life and are downregulated postnatally. *IGF-II* is upregulated in paediatric tumours and developmental syndromes predisposing to such tumours (e.g. Beckwith–Wiedemann syndrome). These factors represent tumour markers as they display a tissue-specific oncofetal pattern of expression.[8] Transgenic mice overexpressing *IGF-II* have a higher risk of developing tumours, including sarcoma, after a long latent period, which suggests that IGF-II functions primarily as a tumour promoter or progression factor in vivo rather than a potent tumour initiator.[9,10] Understanding the biology of these growth factors and their receptors can lead to new therapeutic approaches.

### Discussion

The evaluation of 29 human sarcoma specimens revealed high levels of expression for IGF-I, IGF-II and the type I IGF receptor mRNA, as determined by RT-PCR and in comparison with control cell lines.[11] Analysis of soft tissue sarcoma cells revealed high steady-state levels of the type I IGF receptor mRNA transcripts and protein which correlated with receptor-specific tyrosine kinase activity.[12] Hypophysectomy profoundly inhibits the metastatic behavior of injected RIF-I fibrosarcoma cells in mice, whereas GH administration provokes the occurrence of lung metastasis, and it was concluded that somatostatin analogues, GH or IGF antagonists may suppress metastasis of certain tumours.[13] The presence of IGF-I mRNA in leiomyomas and leiomyosarcomas,[14] and the IGF-I immunoreactivity in leiomyosarcomas (7 out of 8), synovial sarcomas (2/3), liposarcomas (3/6), fibrosarcomas (1/3) and in one angiosarcoma,[15] suggest the potential role for IGF-I in stimulating cell proliferation in these tumours. Contrary to the uniform pattern of IGF-I immunoreactivity seen in other sarcomas, only the spindle cell and not the epithelial component of synovial sarcomas exhibited strong IGF-I immunoreactivity. In malignant fibrous histiocytomas, heterogeneous staining was observed for IGF-I, mostly seen as a diffuse cytoplasmatic reaction in the majority of the tumour cells. A separate study in two noninflammatory fibrous histiocytomas showed also significant immunohistochemical staining for IGF-I.[16] Abundant IGF-II mRNA species have also been detected in histiocytoma tissue.[17]

### *Leiomyomas and leiomyosarcomas*

In normal myometrium and leiomyomas, the *IGF-II* gene is expressed at low levels but it is activated in leiomyosarcomas, whereas the *IGF-I* gene appears repressed in leiomyosarcomas.[18] The expression of a fourth leader exon (*hE4*) which leads to the formation of a 5.0-kb mRNA is enhanced 10-fold in leiomyosarcoma tissue, but it has yet to be established whether there is a causal relation between the activation of *hE4* expression and tumour formation.[19] In normal smooth muscle and in leiomyomas the *IGF-II* gene appeared to be methylated, whereas, in leiomyosarcomas, methylation of DNA was low and it was suggested that there is an inverse correlation between the methylation state and expression of the *IGF-II* gene.[20] Relaxation of *IGF-II* genomic imprinting was also observed in uterine leiomyosarcomata.[21] There was also a correlation between an *Ava*II restriction fragment length polymorphism in the *IGF-II* gene and the occurrence of smooth muscle tumours.[22] More than 90% of liposarcomas exhibit greatly elevated IGF-II mRNA levels, while normal adipose tissue contained very low or undetectable IGF-II levels.[23]

### *Ewing sarcoma/primitive neuroectodermal tumour*

Most neuroectodermal tumour cell lines and tumours with a t(11;22) translocation (primitive neuroectodermal tumour (PNET), Ewing's sarcoma, esthesioneuroblastoma) expressed IGF-I mRNA, whereas none of the cell lines without the translocation (PNET, neuroblastoma) expressed IGF-I mRNA transcripts.[24,25] Data indicate that IGF-I may play an important role for the growth of ES/PNET tumour cells.[26] Loss of imprinting (LOI) of *IGF-II* occurs in some Ewing's sarcomas but is not associated with increased expression of IGF-II mRNA, suggesting that LOI may be related to genetic or epigenetic abnormalities in tumours independent of *IGF-II* expression.[27]

The IGF-I receptor-mediated loop was found to be constantly present in ES/PNET cells and the addition of a specific type I IGF receptor ($\alpha$IR-3) antibody suppressed the growth of ES/PNET cells by decreasing the proliferative rate and increasing apoptosis. Furthermore, the $\alpha$IR-3 antibody significantly inhibited the ability of ES/PNET cells to grow in soft agar and migrate following a chemotactic stimulus.[28] A decrease in the number of type I IGF receptors causes massive apoptosis in several transplantable tumours, whereas if overexpressed the type I IGF receptor protects cells from apoptosis *in vivo*.[29] The IGF-I receptor appears to mediate cellular proliferation and increased transforming ability through its C-terminal domain.[30] Serum-free growth of Ewing's sarcoma cell lines was achieved by supplementing a basal medium containing IGF-I. IGF-I and -II increased DNA synthesis and glucose transport in Ewing's sarcoma cells.[31] Modulation of the IGF system, which appears to constitute an important stimulator of cell growth in neuroectoderm-derived or -related tumours, can be used to enhance the drug sensitivity of the tumour cells in *in vivo* and *in vitro* therapeutic procedures.[32]

*Desmoplastic small round cell tumour*

Desmoplastic small round cell tumour (DSRCT) is an abdominal malignancy in children characterized by a recurrent chromosomal translocation, t(11;22) (p13;q12).[33,34] A genomic DNA fragment containing a Ewing's sarcoma (*EWS*) and a Wilms' tumour (*WT1*) fusion gene has been isolated from these tumours.[35–37] The aberrant EWS/WT1 transcription factor activated the *type I IGF receptor* promoter by approximately 340%, whereas expression vectors encoding either EWS or WT1 reduced the activity of the promoter to 46 and 58% of control values, respectively. Since the EWS/WT1 chimeric protein obtains the three C-terminal zinc fingers of the DNA binding domain of *WT1*, it is possible that this fusion protein may modulate transcription of target genes containing *WT1* binding motifs, such as the *type 1 IGF receptor* gene. Thus, activation of the *IGF-I receptor* promoter by the EWS/WT1 fusion protein may constitute a potential mechanism for the etiology of this particular malignancy.[38] In addition, Ewing's sarcoma cells expressing antisense *EWS* fusion transcripts showed loss of anchorage-independent growth and tumorigenicity in nude mice, which emphasizes the importance of targeting the EWS fusion products as a therapy for the Ewing family of tumours.[39] Interestingly, in the case of the *EWS/FLI-1* fusion protein, the IGF-I receptor is required for the transformation of cells.[40]

*Rhabdoid tumour of the kidney*

Malignant rhabdoid tumour of the kidney (MRTK) showed strong and specific IGF-II mRNA expression by tumour cells.[41,42] Clear cell sarcoma of the kidney expresses *IGF-II* but not *WT1* transcripts, and *in situ* hybridization patterns for *IGF-II* are similar to primitive metanephrogenic blastemal cells and early stromagenic cells.[43] Slot blot hybridization revealed that IGF-II mRNA was only slightly increased in a clear cell sarcoma of the kidney, but was not elevated in two malignant rhabdoid tumours of the kidney.[44]

*Rhabdomyosarcoma*

Recent evidence links abnormal development of the skeletal muscle pathway with rhabdomyosarcoma. The shh/ptc/pax signaling pathway is involved in the induction of myogenic differentiation in somites and in neural tube tissue. A consistant feature of alveolar rhabdomyosarcoma is a translocation involving either a fusion of *Pax-3* or, to a lesser extent, *Pax-7* with another transcription factor *fkhr*.[45] Binding of shh to ptc activates the zinc finger transcription factor *gli-1*, which is frequently amplified in human sarcomas and brain tumours.[46] Mice heterozygous for *ptc* inactivation show a high incidence of rhabdomyosarcoma with overexpression of *ptc*, *gli-1* and *IGF-II* in the tumour, but not in surrounding normal skeletal tissue. This suggests a cross-talk between the ptc and IGF-II signaling pathways in the pathogenesis of rhabdomyosarcoma.[47]

Most rhabdomyosarcomas possess two or more copies of active *IGF-II* alleles, arising either by relaxation of imprinting or duplication of the active allele, whereas in normal muscle monoallelic expression of the *IGF-II* gene is conserved.[48] Of the four RMS heterozygotes, 50% had biallelic expression of *IGF-II*.[49] Furthermore, the imprinting of all *IGF-II* promoters is relaxed in RMS, indicating that loss of imprinting of *IGF-II* gene promoters may be regulated in a coordinated manner by a common mechanism in these tumours.[50] In embryonal RMS, a tumour-suppressor locus has been implicated at chromosome band 11p15.5.[51] Furthermore, there is evidence that this tumour suppressor is imprinted in a manner opposite to that of *IGF-II*.[52] Matsumoto *et al.*[53] concluded that loss of imprinting of *IGF-II* itself might not induce tumour occurrence in tissues where the control of tissue-specific expression of *IGF-II* is maintained, and that increased expression of *IGF-II* due to maternal loss of a putative controller gene for IGF-II at 11p15 might predispose to sustaining tumorigenic mutations and tumour progression, and that loss of a putative onco-suppressor gene at 11p15 might induce RMS occurrence.

*H19* is another possible tumour-suppressor gene for embryonal rhabdomyosarcoma as it is also located at chromosome 11p15.5 and is paternally imprinted.[54,55] Expression of *H19* was observed in four out of six embryonal rhabdomyosarcomas.[56] The expression of the *H19* gene is significantly suppressed as compared to normal muscle tissue in 13 out of 15 rhabdomyosarcomas with embryonal histology, and in three out of 11 rhabdomyosarcomas classified as alveolar subtype. It is evident that the genetic and epigenetic alterations affecting chromosome 11p15 in a high number of RMSs cause deregulation of several imprinted genes, including the extinction of *H19* and an increase in the number of active *IGF-II* alleles, thus eventually leading to tumour growth.[57] On the contrary, cellular growth rates are reduced in *H19* transfected embryonal rhabdomyosarcoma cell line.[58]

Rhabdomyosarcoma, a tumour of skeletal muscle origin, appears developmentally arrested at an early stage in the myogenic differentiation pathway. IGF-II mRNA has previously been shown to be expressed at high levels in RMS.[59,60] In the human RMS cell line IN157, high levels of 6.0-, 4.8- and 4.2-kb IGF-II mRNA transcripts are expressed, whereas normal skeletal muscle expresses negligible amounts of IGF-II mRNA.[61] In addition, this cell line secretes two forms of IGF-II of medium molecular size, 10 and 7.5 kDa, which play a role in the autocrine control of cell growth.[62] A minor 4.8-kb mRNA was exclusively engaged in the synthesis of the prepropeptide on membrane-bound polysomes, whereas a major 6.0-kb mRNA was present in a cytoplasmatic particle, suggesting that translational discrimination between the mRNAs is dictated by their different 5′-untranslated regions.[63] High levels of IGF-II mRNA are found in both alveolar and embryonal RMS.[64–66] IGF-II mRNA expression is limited to tumour cells and is not found in the surrounding stroma, suggesting that an autocrine loop for IGF-II may be functional *in vivo* in RMS.[65] Poorly differentiated RMS showed the highest level of IGF-II mRNA expression, whereas well-differentiated RMS showed low expression, albeit still significantly higher than in normal differentiated skeletal muscle fibers. Thus, IGF-II has potential as a marker for rhabdomyosarcomas and other soft tissue sarcomas that could be especially useful in differential diagnosis of these tumours.[67] Furthermore, *IGF-II* overexpression in myoblasts resulted in an increased proliferative rate, impairment of the ability to differentiate into myoblasts and acquisition of the capability of anchorage-independent growth. It was concluded that *IGF-II* overexpression in muscle myoblasts leads to morphological and biological changes typical of the malignant phenotype, and represents a pivotal event in the pathogenesis of RMS.[68]

The capacity of growth factors to induce a motility response in cells has important implications for the invasive and metastatic potential of tumour cells in particular. IGFs, in particular, have the capacity to stimulate cellular motility in tumour cell lines via different receptors and probably different signal transduction pathways. As a consequence, enhanced motility confers upon the cell an increased metastatic potential.[7] Exogenous IGF-II stimulates cellular motility of rhabdomyosarcoma cell lines via an IGF-I receptor-independent pathway.[69] IGF-II elicits a mitogenic response through the type I IGF receptor and a motility response through the type II IGF receptor.[70]

Suramin is a polysulfonated naphthyl-urea with antineoplastic activity which interferes non-selectively with the binding of growth factors to their cellular receptors. In particular, it displaces [$^{125}$I] IGF-I from the type I IGF receptor, indicating that suramin exerts its effect on RMS cell growth by interrupting the IGF-II autocrine loop in these cells.[71] In the alveolar RMZ-RC2 and the embryonal CCA RMS cells, suramin induces a significant increase in the proportion of myosin-positive cells over control cultures. Thus, suramin both inhibits growth and induces myogenic differentiation.[72]

In RD and HTB114 RMS cell lines which express mutant TP53 protein, transfection with wild-type *TP53* expression vectors led to a reduction in *IGF-II P3 promoter* expression in these cells.[73] Furthermore, TP53 binds to the P4 proximal promoter element which results in the inhibition of P4 activity together with a 5-fold reduction of IGF-II in mRNA derived from the P4 promoter.[74] In RMS cells, tumour-derived forms of TP53 stimulated the activity of the *type I IGF receptor*, suggesting that wild-type TP53 has the potential to suppress the *type I IGF receptor* in the differentiated cell, thus resulting in low levels of receptor gene expression in adult tissues.[75]

Alveolar rhabdomyosarcoma cell lines are very sensitive to the growth-inhibiting effects of the immunosuppressive agent, rapamycin, which inhibits the type I IGF receptor-mediated signalling.[76] A specific type I IGF receptor-blocking antibody ($\alpha$IR-3) suppresses RMS growth *in vivo*. The decrease in tumour growth was associated with a decrease of *p34$^{cdc2}$*, which is involved in cell cycle regulation suggesting that treatment results in the arrest of cellular proliferation.[77] Transfection of a human alveolar RMS cell line with an amplifiable type I IGF receptor antisense expression vector was associated with markedly reduced growth rates in *in vitro*, impaired colony formation in soft agar and a failure of tumour formation in immunodeficient mice.[78] The Rh30 alveolar RMS cells, which are stably transfected with antisense type I IGF receptor, showed significant reduction in growth rate, an increased expression of *MyoD*, *myosin heavy chain*, and an increased number of multinucleated cells in comparison to the parental line. The expression of a *type I IGF receptor* that carries a mutation in the intracellular $\beta$-subunit markedly decreased the response of RMS cells to stimulation with IGF-I and, in addition, resulted in a decrease of RMS growth *in vivo* suggesting that a prospective gene therapy may use this novel strategy to inhibit RMS growth.[79] In addition, recombinant human $\alpha_{2a}$-interferon induced growth arrest in these cells, associated with down-regulation of the *type I IGF receptor*.[80] Over-expression of *type I IGF receptors* and/or *IGF ligands* may thus confer a proliferative advantage on sarcomas over normal adjacent tissues. Treatment of both embryonal and alveolar human RMS cell lines with all-*trans*-retinoic acid resulted in a dose-dependent inhibition of cell growth which is not reversed by addition of exogenous IGF-II.[81] Molecular therapies may also target the signal transduction pathway, in particular the SHC-GRB2 protein complexes and MAP kinases which have been characterized in RMS tumours and cell lines.[82]

Increased circulating levels of IGFBP-2 were found in various neoplastic conditions, including Wilms' tumours[83] and it was concluded that IGFBP-2 measurements might be of value as a marker for monitoring tumour patients during therapy. Serum IGFBP-2 levels were increased in patients with solid peripheral tumours, whereas patients in complete remission had normal IGFBP-2 levels.[84] In culture, the A 673 RMS cell line has been shown to secrete a specific IGFBP found also in the spinal fluid.[85] In media conditioned by RMS cell lines A 673 and RD, leiomyosarcoma cell line

SK-LMS, as well as leiomyoblastoma cell line G 402, IGFBP-2 has been found in large amounts.[86] Whereas IGFBP-3 are synthesized at high levels by the leiomyoblastoma cell line G 402,[86] levels were decreased in leiomyosarcoma sections[87] which may confer a growth advantage upon malignant smooth muscle cells.

### *Non-islet cell tumour hypoglycaemia*

Sarcomas are occasionally associated with the occurrence of hypoglycaemia. Non-islet cell tumours which induce hypoglycaemia are rare. They are usually intra-abdominal or thoracic. The underlying mechanism for the hypoglycaemia is the production of IGF-II, predominantly as a high-molecular weight form ('big' IGF-II), by these tumours.[88,89] The *IGF-II* gene is overexpressed in many mesenchymal tumours, and the levels of 'big' IGF-II are increased in serum from patients with non-islet cell tumour hypoglycaemia (NICTH).[90] In patients with haemangiopericytoma, hypoglycaemia was associated with increased serum levels of 'big' IGF-II.[91,92] The serum of a patient with a large intra-abdominal haemangiopericytoma contained mainly a large-molecular weight precursor IGF-II (mol. wt. 15–20 kDa), which disappeared from the serum after operation.[93] In another patient with a meningeal haemangiopericytoma and a large metastatic liver, hypoglycaemia was associated with low insulin, distinctly decreased IGF-I and normal IGF-II, but high 'big' IGF-II levels.[94] High levels of IGF-II mRNA and IGF-II peptide were detected in both primary meningeal haemangiopericytoma and metastatic foci in the liver.[95] In a patient with pelvic clear cell sarcoma, severe hypoglycaemia linked to elevated production of 'big' IGF-II with acromegaloid swelling returned to normal after tumour resection.[96] A patient with a huge fibrosarcoma in the right liver lobe, associated with hypoglycaemia, became euglycaemic after transcatheter arterial embolization. Interestingly, IGF-II intensely stained in the Golgi area of the tumour cells.[97] In a leiomyosarcoma, high concentrations of IGF-II mRNA and elevated IGF-II immunoreactivity were detected with a 77% fraction of high-molecular weight IGF-II[98] and, in a histiocytoma, a 100-fold elevated IGF-II mRNA level was found as compared to normal liver.[99] Thus, low or normal IGF-II levels are found in serum, despite demonstrable overexpression of IGF-II mRNA by the tumour.[100] It was suggested that abnormal IGF-II binding to the 150-kDa IGFBP may play a role in tumour-associated hypoglycaemia.[101] The pro-IGF-II is, to a large extent, bound to low-molecular weight IGF-BPs which are able to freely exit the vascular compartment and reach target tissues, where the IGF-II may exert its insulin-like activity.[102] Serum IGFBP-3 was also expressed in forms of about 60 kDa instead of the expected size of about 140 kDa.[103] Furthermore, not only suppression of IGFBP-3 but also a 10-fold increase of IGFBP-2 levels has been described.[104]

### Conclusion

There is formidable evidence which supports the notion that IGFs play a pivotal role in human cancer. Relaxation of *IGF-II* genomic imprinting occurs in childhood as well as adult-onset tumours, and may thus represent a novel epigenetic mechanism for oncogenesis throughout life. The overexpression of the *type I IGF receptor* renders the cells susceptible to transformation by oncogenes. Thus, attempts are currently being made to inhibit cell proliferation by targeting the IGF-I receptor, by means of anti-receptor antibodies, IGF analogues or antisense strategies, with the common goal of inducing apoptosis of neoplastic cells.[105] These therapeutic possibilities do offer an intruiging scenario to be developed further in an effort to provide patients affected with different kinds of soft tissue sarcomas with a better outcome.

### Acknowledgement

I wish to express my gratitude to Dr. Paul N. Schofield, Department of Anatomy, University of Cambridge, England, for his kind support and stimulating discussions. The support of the Kinderkrebshilfe Heidelberg is also gratefully acknowledged.

### References

1 Werner H, LeRoith D. The role of the insulin-like growth factor system in human cancer. *Adv Cancer Res* 1996; 68:183–223.
2 Wu X, Fan Z, Masui H, *et al.* Apoptosis induced by blocking epidermal growth factor receptors is rescued by insulin through insulin-like growth factor I receptor. *Proc Annu Meet Am Assoc Cancer Res* 1994; 35:A16.
3 Sell C, Baserga R, Rubin R. Insulin-like growth factor I (IGF-I) and the IGF-I receptor prevent etoposide-induced apoptosis. *Cancer Res* 1995; 55:303–6.
4 Sporn MB, Roberts AB. Autocrine growth factors and cancer. *Nature* 1985; 313:745–7.
5 Zumkeller W, Schofield PN. Growth factors, cytokines and soluble forms of receptor molecules in cancer patients. *Anticancer Res* 1995; 15:344–8.
6 Zumkeller W, Groth O, Commentz J. Regulation of insulin-like growth factors and IGF-binding proteins in bone tumours. *Growth Regul* 1996; 6:10–15.
7 Werner H, LeRoith D. The role of the insulin-like growth factor system in human cancer. *Adv Cancer Res* 1996; 68:183–223.
8 Biran H, Ariel I, de Groot N, *et al.* Human imprinted genes as oncodevelopmental markers. *Tumour Biol* 1994; 15:123–34.
9 Rogler CE, Yang D, Rossetti L, *et al.* Altered body composition and increased frequency of diverse malignancies in insulin-like growth factor-II transgenic mice. *J Biol Chem* 1994; 269:13779–84.

10 Bates P, Fisher R, Ward A, *et al.* Mammary cancer in transgenic mice expressing insulin-like growth factor II (IGF-II). *Br J Cancer* 1995; 72:1189–93.
11 Sekyi-Otu A, Bell RS, Ohashi C, *et al.* Insulin-like growth factor 1 (IGF-1) receptors, IGF-1, and IGF-2 are expressed in primary human sarcomas. *Cancer Res* 1995; 55:129–34.
12 Beech D, Pollock RE, Tsan R, *et al.* Epidermal growth factor receptor and insulin-like growth factor-I receptor expression and function in human soft-tissue sarcoma cells. *Int J Oncol* 1998; 12:329–36.
13 Sekyi-Otu A, Bell RS, Andrulis IL, *et al.* Metastatic behaviour of RIF-1 murine fibrosarcoma: inhibited by hypophysectomy and partially restored by growth hormone replacement. *J Natl Cancer Inst* 1994; 86:628–32.
14 Höppener J, Mosselman S, Roholl PJ, *et al.* Expression of insulin-like growth factor I and II genes in human smooth muscle tumours. *EMBO J* 1988; 7:1379–85.
15 Roholl PJ, Skottner A, Prinsen I, *et al.* Expression of insulin-like growth factor 1 in sarcomas. *Histopathology* 1990; 16:455–60.
16 Melhem MF, Meisler AI, Saito R, *et al.* Cytokines in inflammatory malignant fibrous histiocytoma presenting with leukemoid reaction. *Blood* 1993; 82:2038–44.
17 Ikejiri K, Wasada T, Haruki K, *et al.* Identification of a novel transcription unit in the human insulin-like growth factor-II gene. *Biochem J* 1991; 280:439–44.
18 Gloudemans T, Prinsen I, Van Unnik JAM, *et al.* Insulin-like growth factor gene expression in human smooth muscle tumours. *Cancer Res* 1990; 50:6689–95.
19 Holthuizen P, van der Lee FM, Ikejiri K, *et al.* Identification and initial characterization of a fourth leader exon and promoter of the human IGF-II gene. *Biochim Biophys Acta* 1990; 1087:341–3.
20 Gloudemans T, Pospiech I, van der Ven LT, *et al.* Expression and CpG methylation of the insulin-like growth factor II gene in human smooth muscle tumors. *Cancer Res* 1992; 52:6516–21.
21 Vu TH, Yballe C, Boonyanit S, *et al.* Insulin-like growth factor II in uterine smooth muscle tumors: maintenance of genomic imprinting in leiomyomata and loss of imprinting in leiomyosarcomata. *J Clin Endocrinol Metab* 1995; 80:1670–6.
22 Gloudemans T, Pospiech I, van der Ven LT, *et al.* An *ava*II restriction fragment length polymorphism in the insulin-like growth factor II gene and the occurrence of smooth muscle tumors. *Cancer Res* 1993; 53:5754–8.
23 Tricoli JV, Rall LB, Karakousis CP, *et al.* Enhanced levels of insulin-like growth factor messenger RNA in human colon carcinomas and liposarcomas. *Cancer Res* 1986; 46:6169–73.
24 Yee D, Paik S, Lebovic GS, *et al.* Analysis of insulin-like growth factor I gene expression in malignancy: evidence for a paracrine role in human breast cancer. *Mol Endocrinol* 1989; 3:509–17.
25 Yee D, Favoni RE, Lebovic GS, *et al.* Insulin-like growth factor I expression by tumors of neuroectodermal origin with the t(11;22) chromosomal translocation. A potential autocrine growth factor. *J Clin Invest* 1990; 86:1806–14.
26 Hamilton G, Mallinger R, Hofbauer S, *et al.* The monoclonal HBA-71 antibody modulates proliferation of thymocytes and Ewing's sarcoma cells by interfering with the action of insulin-like growth factor I. *Thymus* 1991; 18:33–41.
27 Zhan S, Shapiro DN, Helman LJ. Loss of imprinting of IGF2 in Ewing's sarcoma. *Oncogene* 1995; 11:2503–7.
28 Scotlandi K, Benini S, Sarti M, *et al.* Insulin-like growth factor I receptor-mediated circuit in Ewing's sarcoma/peripheral neuroectodermal tumor: a possible therapeutic target. *Cancer Res* 1996; 56:4570–4.
29 Resnicoff M, Abraham D, Yutanawiboonchai W, *et al.* The insulin-like growth factor I receptor protects tumor cells from apoptosis in vivo. *Cancer Res* 1995; 55:2463–9.
30 Esposito DL, Blakesley VA, Koval AP, *et al.* Tyrosine residues in the C-terminal domain of the insulin-like growth factor-I receptor mediate mitogenic and tumorigenic signals. *Endocrinology* 1997; 138:2979–88.
31 van Valen F, Winkelmann W, Jürgens H. Type I and II insulin-like growth factor receptors and their function in human Ewing's sarcoma cells. *J Cancer Res Clin Oncol* 1992; 118:269–75.
32 Hofbauer S, Hamilton G, Theyer G, *et al.* Insulin-like growth factor-I-dependent growth and in vitro chemosensitivity of Ewing's sarcoma and peripheral primitive neuroectodermal tumour cell lines. *Eur J Cancer* 1993; 29A:241–5.
33 Shen WP, Towne B, Zadeh TM. Cytogenetic abnormalities in an intraabdominal desmoplastic small cell tumor. *Cancer Genet Cytogenet* 1993; 64:189–91.
34 Biegel JA, Conard K, Brooks JJ. Translocation (11;22) (p13;q12): primary change in intra-abdominal desmoplastic small round cell tumor. *Genes Chromosomes Cancer* 1993; 7:119–21.
35 Rauscher FJ III, Benjamin LE, Fredericks WJ, *et al.* Novel oncogenic mutations in the WT1 Wilms' tumor suppressor gene: a t(11; 22) fuses the Ewing's sarcoma gene, EWS1, to WT1 in desmoplastic small round cell tumor. *Cold Spring Harbor Symp Quant Biol* 1994; 59:137–46.
36 Ladanyi M, Gerald W. Fusion of the EWS and WT1 genes in the desmoplastic small round cell tumor. *Cancer Res* 1994; 54:2837–40.
37 Gerald WL, Rosai J, Ladanyi M. Characterization of the genomic breakpoint and chimeric transcripts in the EWS-WT1 gene fusion of desmoplastic small round cell tumor. *Proc Natl Acad Sci USA* 1995; 92:1028–32.
38 Karnieli E, Werner H, Rauscher FJ 3rd, *et al.* The IGF-I receptor gene promoter is a molecular target for the Ewing's sarcoma-Wilms' tumor 1 fusion protein. *J Biol Chem* 1996; 271:19304–9.
39 Ouchida M, Ohno T, Fujimura Y, *et al.* Loss of tumorigenicity of Ewing's sarcoma cells expressing antisense RNA to EWS-fusion transcripts. *Oncogene* 1995; 11:1049–54.
40 Toretsky JA, Kalebic T, Blakesley V, *et al.* The insulin-like growth factor-I receptor is required for EWS/FLI-1 transformation of fibroblasts. *J Biol Chem* 1997; 272:30822–7.
41 Gansler T, Gerald W, Anderson G, *et al.* Characterization of a cell line derived from rhabdoid tumor of the kidney. *Hum Pathol* 1991; 22:259–66.
42 Sharifah NA, Yun K. Malignant rhabdoid tumor of the kidney expresses insulin-like growth factor II transcripts. *Pathology* 1994; 26:134–7.
43 Yun K. Clear cell carcinoma of the kidney expresses insulin-like growth factor-II but not WT1 transcripts. *Am J Pathol* 1993; 142:39–47.
44 Briner J, Hassam S, Rohrer R. Nachweis der unterschiedlichen Expression von IGF-II bei verschiedenen embryonalen Nierentumoren durch slot-blot-Hybridisierung und in-situ-Hybridisierung. *Verh Dtsch Ges Pathol* 1990; 74:419–23.
45 Zhan S, Helman LJ. *Gli* the cause of rhabdomyosarcoma. *Nature Med* 1998; 4:559–60.

46 Kinzler KW, Vogelstein B. The GLI gene encodes a nuclear protein which binds specific sequences in the human genome. *Mol Cell Biol* 1990; 10:634–42.
47 Hahn H, Wojnowski L, Zimmer AM, *et al.* Rhabdomyosarcomas and radiation hypersensitivity in a mouse model of Gorlin syndrome. *Nature Med* 1998; 4:619–22.
48 Pedone PV, Tirabosco R, Cavazzana AO, *et al.* Mono- and biallelic expression of insulin-like growth factor II gene in human muscle tumors. *Hum Mol Genet* 1994; 3:1117–21.
49 McCann AH, Miller N, O'Meara A, *et al.* Biallelic expression of the IGF2 gene in human breast disease. *Hum Mol Genet* 1996; 5:1123–7.
50 Zhan S, Shapiro D, Zhan S, *et al.* Concordant loss of imprinting of the human insulin-like growth factor II gene promoters in cancer. *J Biol Chem* 1995; 270:27983–6.
51 Loh WE Jr, Scrable HJ, Livanos E, *et al.* Human chromosome 11 contains two different growth suppressor genes for embryonal rhabdomyosarcoma. *Proc Natl Acad Sci USA* 1992; 89:1755–9.
52 Scrable H, Cavenee W, Ghavimi F, *et al.* A model for embryonal rhabdomyosarcoma tumorigenesis that involves genome imprinting. *Proc Natl Acad Sci USA* 1989; 86:7480–4.
53 Matsumoto T, Kinoshita E, Maeda H, *et al.* Molecular analysis of a patient with Beckwith-Wiedemann syndrome, rhabdomyosarcoma and renal cell carcinoma. *Jpn J Hum Genet* 1994; 39:225–34.
54 Rachmilewitz J, Goshen R, Ariel I, *et al.* Parental imprinting of the human H19 gene. *FEBS Lett* 1992; 309:25–8.
55 Rainier S, Johnson LA, Dobry CJ, *et al.* Relaxation of imprinted genes in human cancer. *Nature* 1993; 362:747–9.
56 Ariel I, Ayesh S, Perlman EJ, *et al.* The product of the imprinted H19 gene is an oncofetal RNA. *J Clin Pathol: Mol Pathol* 1997; 50:34–44.
57 Casola S, Pedone PV, Cavazzana AO, *et al.* Expression and parental imprinting of the H19 gene in human rhabdomyosarcoma. Oncogene 1997; 14:1503–10.
58 Hao Y, Crenshaw T, Moulton T, *et al.* Tumour-suppressor activity of H19 RNA. *Nature* 1993; 365:764–7.
59 Scott J, Cowell JK, Robertson ME, *et al.* Insulin-like growth factor-II gene expression in Wilms' tumour and embryonic tissues. *Nature* 1985; 317:260–2.
60 Gray A, Tam AW, Dull TJ, *et al.* Tissue-specific and developmentally regulated transcription of the insulin-like growth factor 2 gene. *DNA (NY)* 1987; 6:283–95.
61 Nielsen FC, Haselbacher G, Christiansen J, *et al.* Biosynthesis of 10 kDa and 7.5 kDa insulin-like growth factor II in a human rhabdomyosarcoma cell line. *Mol Cell Endocrinol* 1993; 93:87–95.
62 Gammeltoft S, Christiansen J, Nielsen FC, *et al.* Insulin-like growth factor II: complexity of biosynthesis and receptor binding. *Adv Exp Med Biol* 1991; 293:31–44.
63 Nielsen FC, Gammeltoft S, Christiansen J. Translational discrimination of mRNAs coding for human insulin-like growth factor II. *J Biol Chem* 1990; 265:13431–4.
64 Minniti CP, Helman LJ. IGF-II in the pathogenesis of rhabdomyosarcoma: a prototype of IGFs involvement in human tumorigenesis. *Adv Exp Med Biol* 1993; 343:327–43.
65 Minniti CP, Tsokos M, Newton WA Jr, *et al.* Specific expression of insulin-like growth factor-II in rhabdomyosarcoma tumor cells. *Am J Clin Pathol* 1994; 101:198–203.
66 Zhan S, Shapiro DN, Helman LJ. Activation of an imprinted allele of the insulin-like growth factor II gene implicated in rhabdomyosarcoma. *J Clin Invest* 1994; 94:445–8.
67 Yun K. A new marker for rhabdomyosarcoma. Insulin-like growth factor II. *Lab Invest* 1992; 67:653–64.
68 Minniti CP, Luan D, O'Grady C, *et al.* Insulin-like growth factor II overexpression in myoblasts induces phenotypic changes typical of the malignant phenotype. *Cell Growth Diff* 1995; 6:263–9.
69 El Badry OM, Minniti C, Kohn EC, *et al.* Insulin-like growth factor II acts as an autocrine growth and motility factor in human rhabdomyosarcoma tumors. *Cell Growth Diff* 1990; 1:325–31.
70 Minniti CP, Kohn EC, Grubb JH, *et al.* The insulin-like growth factor II (IGF/II)/mannose 6-phosphate receptor mediates IGF-II-induced motility in human rhabdomyosarcoma cells. *J Biol Chem* 1992; 267:9000–4.
71 Minniti CP, Maggi M, Helman LJ. Suramin inhibits the growth of human rhabdomyosarcoma by interrupting the insulin-like growth factor II autocrine growth loop. *Cancer Res* 1992; 52:1830–5.
72 De Giovanni C, Melani C, Nanni P, *et al.* Redundancy of autocrine loops in human rhabdomyosarcoma cells: induction of differentiation by suramin. *Br J Cancer* 1995; 72:1224–9.
73 Zhang L, Kashanchi F, Zhan Q, *et al.* Regulation of insulin-like growth factor II P3 promotor by p53: a potential mechanism for tumorigenesis. *Cancer Res* 1996; 56:1367–73.
74 Zhang L, Zhan Q, Zhan S, *et al.* p53 regulates human insulin-like growth factor II gene expression through active P4 promoter in rhabdomyosarcoma cells. *DNA Cell Biol* 1998; 17:125–31.
75 Werner H, Karnieli E, Rauscher FJ, *et al.* Wild-type and mutant p53 differentially regulate transcription of the insulin-like growth factor I receptor gene. *Proc Natl Acad Sci USA* 1996; 93:8318–23.
76 Dilling MB, Dias P, Shapiro DN, *et al.* Rapamycin selectively inhibits the growth of childhood rhabdomyosarcoma cells through inhibition of signaling via the type I insulin-like growth factor receptor. *Cancer Res* 1994; 54:903–7.
77 Kalebic T, Tsokos M, Helman LJ. In vivo treatment with antibody against IGF-I receptor suppresses growth of human rhabdomyosarcoma and down-regulates $p34^{cdc2}$. *Cancer Res* 1994; 54:5531–4.
78 Shapiro DN, Jones BG, Shapiro LH, *et al.* Antisense-mediated reduction in insulin-like growth factor-I receptor expression suppresses the malignant phenotype of a human alveolar rhabdomyosarcoma. *J Clin Invest* 1994; 94:1235–42.
79 Kalebic T, Blakesley V, Slade C, *et al.* Expression of a kinase-deficient IGF-I-R suppresses tumorigenicity of rhabdomyosarcoma cells constitutively expressing a wild type IGF-I-R. *Int J Cancer* 1998; 76:223–7.
80 Thulasi R, Dias P, Houghton PJ, *et al.* Alpha 2a-interferon-induced differentiation of human alveolar rhabdomyosarcoma cells: correlation with down-regulation of the insulin-like growth factor type I receptor. *Cell Growth Diff* 1996; 7:531–41.
81 Crouch GD, Helman LJ. All-*trans*-retinoic acid inhibits the growth of human rhabdomyosarcoma cell lines. *Cancer Res* 1991; 51:4882–7.
82 McManus MJ, Hutt PJ, Maihle NJ. Phosphotyrosyl proteins in childhood rhabdomyosarcomas: phosphorylation of catenins and components of the insulin-like growth factor type I receptor signalling cascade. *J Pediatr Hematol Oncol* 1997; 19:319–26.

83 Zumkeller W, Schwander J, Mitchell CD, *et al.* Insulin-like growth factor (IGF)-I, -II and IGF binding protein-2 (IGFBP-2) in the plasma of children with Wilms' tumour. *Eur J Cancer* 1993; 29A:1973–7.
84 Müller HL, Oh Y, Lehrnbecher T, *et al.* Insulin-like growth factor binding protein-2 concentrations in cerebrospinal fluid and serum of children with malignant solid tumors or acute leukemia. *J Clin Endocrinol Metab* 1994; 79:428–34.
85 Romanus JA, Tseng LY, Yang YW, *et al.* The 34 kilodalton insulin-like growth factor binding proteins in human cerebrospinal fluid and the A673 rhabdomyosarcoma cell line are human homologues of the rat BRL-3A binding protein. *Biochem Biophys Res Commun* 1989; 163:875–81.
86 Koscielniak EB, Gutbrod R, Blum W, *et al.* Varying patterns of secretion of insulin-like growth factors (IGFs) and their binding proteins (BPs) by sarcoma cell lines. *Proc Am Assoc Cancer Res.* 1994; 35:44.
87 van der Ven LT, van Buul-Offers SC, Gloudemans T, *et al.* Modulation of insulin-like growth factor (IGF) action by IGF-binding proteins in normal, benign, and malignant smooth muscle tissues. *J Clin Endocrinol Metab* 1996; 81:3629–35.
88 Lowe WL Jr, Roberts CT Jr, LeRoith D, *et al.* Insulin-like growth factor-II in nonislet cell tumors associated with hypoglycemia: increased levels of messenger ribonucleic acid. *J Clin Endocrinol Metab* 1989; 69:1153–9.
89 Schofield PN, Nyström A, Smith J, *et al.* Expression of a high molecular weight form of insulin-like growth factor II in a Beckwith-Wiedemann syndrome associated adrenocortical adenoma. *Cancer Lett* 1995; 94:71–7.
90 Daughaday WH, Trivedi B, Baxter RC. Serum 'big insulin-like growth factor II' from patients with tumor hypoglycemia lacks normal E-domain O-linked glycosylation, a possible determinant of normal propeptide processing. *Proc Natl Acad Sci USA* 1993; 90:5823–7.
91 Daughaday WH, Emanuele MA, Brooks MH, *et al.* Synthesis and secretion of insulin-like growth factor II by a leiomyosarcoma with associated hypoglycemia. *N Engl J Med* 1988; 319:1434–40.
92 Chung J, Henry RR. Mechanisms of tumor-induced hypoglycemia with intraabdominal hemangiopericytoma. *J Clin Endocrinol Metab* 1996; 81:919–25.
93 Höög A, Sandberg-Nordqvist AC, Hulting AL, *et al.* High molecular weight IGF-2 expression in a haemangiopericytoma associated with hypoglycemia. *APMIS* 1997; 105:469–82.
94 Cotterill AM, Holly JM, Davies SC, *et al.* The insulin-like growth factors and their binding proteins in a case of non-islet-cell tumour-associated hypoglycaemia. J Endocrinol 1991; 131:303–11.
95 Wegmann W, Vonesch HJ, Kamber J, *et al.* Rezidivierendes und metastasierendes Hämangioperizytom der Meningen mit paraneoplastischer Hypoglykämie. *Schweiz Med Wochenschr* 1994; 124:146–51.
96 Sohda T, Yun K. Insulin-like growth factor II expression in primary meningeal hemangiopericytoma and its metastasis to the liver accompanied by hypoglycemia. *Hum Pathol* 1996; 27:858–61.
97 Trivedi N, Mithal A, Sharma AK, *et al.* Non-islet cell tumour induced hypoglycemia with acromegaloid facial and acral swelling. *Clin Endocrinol* 1995; 42:433–5.
98 Kotani K, Tsuji M, Oki A, *et al.* IGF-II producing hepatic fibrosarcoma associated with hypoglycemia. *Intern Med* 1993; 32:897–901.
99 Wasada T, Hizuka N, Yamamoto M, *et al.* An insulin-like growth factor II-producing histiocytoma associated with hypoglycemia: analysis of the peptide, its gene expression, and glucose transporter isoforms. *Metabolism* 1992; 41:310–6.
100 Schofield PN, Connor H, Turner RC, *et al.* Tumour hypoglycaemia: raised tumour IGFII mRNA associated with reduced plasma somatomedin. *Br J Cancer* 1989; 60:661–3.
101 Daughaday WH, Kapadia M. Significance of abnormal serum binding of insulin-like growth factor II in the development of hypoglycemia in patients with non-islet-cell tumors. *Proc Natl Acad Sci USA* 1989; 86:6778–82.
102 Baxter RC. The role of insulin-like growth factors and their binding proteins in tumor hypoglycemia. *Horm Res* 1996; 46:195–201.
103 Baxter RC, Daughaday WH. Impaired formation of the ternary insulin-like growth factor-binding protein complex in patients with hypoglycemia due to nonislet cell tumors. *J Clin Endocrinol Metab* 1991; 73:696–702.
104 Baxter RC, Holman SR, Corbould A, *et al.* Regulation of the insulin-like growth factors and their binding proteins by glucocorticoid and growth hormone in nonislet cell tumor hypoglycemia. *J Clin Endocrinol Metab* 1995; 80:2700–8.
105 Baserga R. The insulin-like growth factor I receptor: a key to tumor growth? *Cancer Res* 1995; 55:249–52.
